# PEG-Poly(1-Methyl-l-Tryptophan)-Based Polymeric Micelles as Enzymatically Activated Inhibitors of Indoleamine 2,3-Dioxygenase

**DOI:** 10.3390/nano9050719

**Published:** 2019-05-09

**Authors:** George Lo Huang, Anqi Tao, Takuya Miyazaki, Thahomina Khan, Taehun Hong, Yasuhiro Nakagawa, Horacio Cabral

**Affiliations:** Department of Bioengineering, Graduate School of Engineering, The University of Tokyo, 7-3-1 Hongo, Bunkyo-ku, Tokyo 113-8656, Japan; huang@bmw.t.u-tokyo.ac.jp (G.H.); tao@bmw.t.u-tokyo.ac.jp (A.T.); tmiyazaki@bmw.t.u-tokyo.ac.jp (T.M.); khan@bmw.t.u-tokyo.ac.jp (T.K.); hong@bmw.t.u-tokyo.ac.jp (T.H.); nakagawa@bmw.t.u-tokyo.ac.jp (Y.N.)

**Keywords:** polymeric micelles, indoleamine 2,3-dioxygenase, 1-Methyl-Tryptophan, enzymatic degradation, immunotherapy

## Abstract

Indoleamine 2,3-dioxygenase (IDO) is an immunomodulating enzyme that is overexpressed in many cancers with poor prognosis. IDO suppresses T cell immunity by catabolizing tryptophan into kynurenine (KYN), which induces apoptosis in T effector cells and enhances T regulatory cells, providing a powerful immunosuppressive mechanism in tumors. Thus, major efforts for developing IDO inhibitors have been undertaken. Among them, 1-Methyl-l-Tryptophan (MLT) and 1-Methyl-d-Tryptophan (MDT) effectively inhibit IDO in preclinical tumor models and the latter is under clinical evaluation. However, both MLT and MDT present poor pharmacokinetics, with the maximum serum concentration being below their 50% inhibitory concentration value. Herein, we have developed polymeric IDO inhibitors based on MLT, which can release active MLT after enzymatic degradation, toward establishing superior antitumor immunotherapies. These polymers were prepared by ring opening polymerization of an N-phenyl carbamate (NPC) derivative of MLT that was synthesized by carbamylation with diphenyl carbonate. By using ω-amino-poly(ethylene glycol) (PEG-NH_2_) as the macroinitiator, we prepared amphiphilic PEG-poly(MLT) block copolymers, which self-assembled into polymeric micelles in aqueous conditions. The PEG-poly(MLT) block copolymers could be readily degraded by chymotrypsin and the micelles were able to reduce the levels of KYN in activated macrophages. These results provide a strong rationale for pursuing MLT-based polymeric micelles as tumor-targeted prodrug systems.

## 1. Introduction

Immune therapy is an exciting approach for treating cancer that can generate strong responses in a limited population of cancer patients. Since the approval of ipilimumab in 2011 to treat melanoma, 11 new immunotherapies have been approved and have quickly become the standard of care for many cancer types [1]. Although response rates in approved immunotherapies are impressive, a significant number of cancer patients do not respond well to immune therapy. Tumor mediated immunosuppression is believed to be the main culprit for poor response to immune therapy and there is great academic and clinical interest in reversing tumor mediated immunosuppression to increase the response rate and improve outcomes of existing and approved immunotherapies [1,2]. 

**Figure 1:**
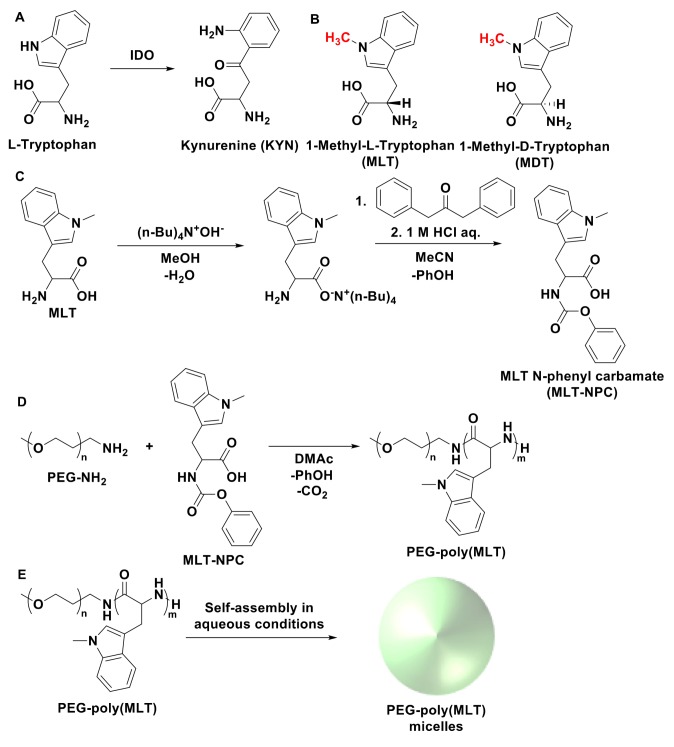
(**A**) Indoleamine 2,3-dioxygenase (IDO) catabolizes L-tryptophan into Kynurenine, leading to immunosuppression. (**B**) Two IDO inhibitor homologs, 1-Methyl-l-Tryptophan (MLT) and 1-Methyl-d-Tryptophan (MDT; Indoximod). (**C**) Synthesis scheme of MLT-N-phenyl carbamate (NPC). (**D**) Ring opening polymerization of MLT-NPC with polyethylene glycol initiator. (**E**) Self-assembly of block copolymer into micelle in aqueous conditions.

Indoleamine 2,3-dioxygenase (IDO), is an enzyme that is overexpressed in many cancers and is implicated in tumor mediated immunosuppression [3,4,5]. IDO functions by catabolizing tryptophan into kynurenine (KYN) (Figure 1A), causing local tryptophan depletion, which induces anergy and apoptosis in effector T cells [5,6]. Additionally, KYN can encourage naïve T cell differentiation into regulatory T cells. Through tryptophan metabolism, IDO induces immunosuppression through regulation of T cell populations in the cancer microenvironment. As IDO is expressed in many cancers that are resistant to immunotherapy [5,7], one proposed method to enhance currently approved immunotherapies is to inhibit IDO activity in tumors through small molecule inhibitors. The methylated tryptophan derivatives, i.e., 1-Methyl-l-Tryptophan (MLT) and 1-Methyl-d-Tryptophan (MDT) (Figure 1B), have shown IDO inhibitory activities in preclinical studies [8,9,10] and MDT is currently in phase III clinical trials under the name indoximod [8]. Nevertheless, both drugs present poor bioavailability, which significantly limits their potential efficacy [6]. For example, MDT is orally dosed at the maximum intestinal absorption limit, i.e., 2 g per day, though its maximum detectable serum concentration (C_max_) is only 12 µM [6], which is lower than the 50% inhibitory concentration (IC_50_) of MDT, i.e., 20 µM [9]. This situation denotes that therapeutic concentrations of MDT cannot be achieved within the tumoral microenvironment and suggests that approaches capable of increasing the drug levels in tumors could have high potential for enhancing the efficacy.

A possible method to overcome the poor pharmacokinetics of MLT and MDT is to formulate tumor-targeted drug delivery systems (DDS) for enhancing the drug levels in tumors [10,11,12]. However, because of the high dosage requirements for MLT and MDT, the development of traditional DDS, such as liposomes or polymer-drug conjugates, could be rendered unrealistic. Therefore, herein, we hypothesized that developing polymeric forms of MLT and MDT could be a viable approach for enhancing the pharmacokinetics and the drug levels in tumors toward superior efficacy. Thus, both MLT and MDT could be used as building units of polymers having peptide backbones, which may be cleaved by enzymes to release active IDO inhibitors. These polymers would also be hydrophobic, allowing for the synthesis of amphiphilic block copolymers in combination with hydrophilic polymers like poly(ethylene glycol) (PEG). In aqueous conditions, such block copolymers would assemble into core-shell polymeric micelles [11,12], which have demonstrated tremendous potential as nanocarriers against cancer by increasing the delivery of drugs into tumor tissues based on the increased permeability of tumor blood vessels and the impaired lymphatic drainage in tumor tissues [13]. These polymers could have dual functionality as polymer-drug conjugates, but also as a drug delivery vehicle for carrying other anticancer therapeutics. Thus, in this study, after assessing the ability of enzymes to cleave poly(MLT) (P(MLT)) and poly(MDT) (P(MDT)), we developed a polymerization approach for efficient synthesis of PEG-P(MLT) block copolymers and assembled polymeric micelles. In addition, the biological activity of these micelles was validated in activated macrophages, with particular focus on their capability of inhibiting KYN production.

## 2. Materials and Methods

### 2.1. Materials

Unless otherwise indicated, solvents and reagents were used without further purification. DMTMM, MLT and MDT were purchased from Sigma (St. Louis, MO, USA). Chymotrypsin and diphenyl carbonate were purchased from Tokyo Chemical Industry Co., Ltd. (Tokyo, Japan). α-Methoxy-ω-amino-poly(ethylene glycol) (MeO-PEG-NH_2_; *M_w_* = 2000) was purchased from NOF Co., Inc. (Tokyo, Japan). Spectra/Por dialysis tube (molecular weight cut-off (MWCO) = 1000 Da) was purchased from Spectrum Laboratories (Rancho Dominguez, CA, USA).

### 2.2. Cell Lines

HEK 293 human embryonic kidney cells and RAW 264.7 murine macrophage cells were acquired from the JCRB Cell Bank (Tokyo, Japan) were cultured in DMEM, supplemented with 10% FBS, 1% penicillin and streptomycin and maintained at 37 °C under 5% CO_2_. THP-1 human monocytes were acquired from the JCRB Cell Bank (Tokyo, Japan) and cultured in RPMI-1640, supplemented with 10% FBS, 1% penicillin and streptomycin and maintained at 37 °C under 5% CO_2_.

### 2.3. Synthesis of P(MLT) and P(MDT) by Condensation Reaction

To a stirred suspension of MLT and MDT (1 g, 4.5 mmol) in water (10 mL), NaOH (1 M) was added dropwise at room temperature until fully dissolved. The resulting solutions were then diluted with DMF (10 mL). DMTMM (1.25 g, 4.5 mmol) was dissolved in water (2 mL) and added dropwise to MLT and MDT solutions and left stirring for 24 h. The products were then precipitated in water (1 L) and filtered and dried under vacuum. The products were analyzed by GPC to confirm the presence of polymers and by HPLC to confirm no remaining methyl tryptophan monomers.

### 2.4. Enzymatic Digestion of P(MLT) and P(MDT)

MLT and MDT homopolymers (10 mg) were dissolved in NMP (1 mL). Chymotrypsin hydrochloride (20 mg) was dissolved in HEPES buffer (2 mL). Chymotrypsin solution (1 mL) was added to polymer solutions and transferred to dialysis bags (MWCO = 1000 Da) and dialyzed against 15 mM HEPES buffer (50 mL) containing 10 mM of CaCl_2_ and a 1% solution of penicillin and streptomycin [14]. Polymers were shaken at 37 °C for 48 h and analyzed by HPLC to quantify free methyl tryptophan released.

### 2.5. Synthesis of MLT-NPC

To a stirred suspension of MLT (1 g, 4.5 mmol) in methanol (10 mL), tetrabutylammonium hydroxide (1 M in methanol) (4.5 mL, 4.5 mmol) was added dropwise at room temperature. After stirring for 1 h, methanol was removed by rotary evaporation to leave an oily residue [15]. The resulting residue was dissolved in acetonitrile (10 mL) and the resulting solution was added dropwise to a stirred solution of diphenyl carbonate (1 g, 4.5 mmol) in acetonitrile (10 mL) at room temperature and was left stirring for 3 h. Distilled water (25 mL) was added to the solution and the resulting mixture was acidified to pH 2–3 with 1 M HCl and extracted with ethyl acetate (3 × 15 mL). The combined organic layers were dried over MgSO_4_, filtered and concentrated by rotary evaporation. The crude products were purified by column chromatography (eluting with a gradient from 25% to 75% ethyl acetate in *n*-hexane). The resulting eluent was concentrated by rotary evaporation and precipitated in cold hexane to give 0.8 g (51%) of MLT-NPC as an off-white powder. The products were confirmed by ^1^H-NMR and ^13^C-NMR by using a JEOL ECS-400 spectrometer (JEOL Ltd., Tokyo, Japan) and the chemical shifts were recorded in ppm units using tetramethyl silane (TMS) as an internal standard. ^1^H NMR (400 MHz, dimethyl sulfoxide, DMSO-d_6_, ppm): 3.05–3.17 (m, 1H), 3.69–3.83 (m, 3H), 4.22–4.30 (m, 1H), 6.92–7.45 (m, 9H), 7.50–7.60 (d, 1H), 8.08 (d, 1H).) ^13^C NMR (100 MHz, DMSO-d_6_, d, ppm): 27.31, 32.84, 55.79, 109.94, 110.15, 118.97, 119.07, 121.62, 122.09, 125.50, 128.06, 128.63, 129.77, 137.13, 151.38, 154.81, 173.73. Products were also analyzed by MALDI-TOF by using a JMS-S3000 spectrometer (JEOL Ltd., Tokyo, Japan) to confirm the molecular weight.

### 2.6. Synthesis of PEG-P(MLT) Block Copolymer

PEG-P(MLT) was prepared by the ring opening polymerization of MLT-NPC. The reaction was initiated by the terminal amino group of α-methoxy-ω-amino PEG. The PEG macro-initiator (20 mg; 0.01 mmol; 2000 *M_w_*) was dissolved in 2 mL of anhydrous DMAc. MLT-NPC (70 mg; 0.2 mmol) was dissolved in a solution of 1 mL anhydrous DMAc and added to the solution of PEG macro-initiator. The reaction mixture was stirred for 24 h at 60 °C under argon atmosphere. The resulting polymer was precipitated into diethyl ether (500 mL). The molecular weight distribution of the polymer was characterized by gel permeation chromatography (column: TSK-gel G4000HHR (Tosoh Co., Inc., Yamaguchi, Japan); eluent: DMF containing 10 mM LiCl; flow rate: 0.8 mL/min; detector: refractive index (RI); temperature: 25 °C). The polymer was also analyzed by ^1^H-NMR (400 MHz, DMF-d_7_, ppm): 3.45–3.82, 4.00–5.00, 6.63–7.76.

### 2.7. Enzymatic Digestion of PEG-P(MLT)

PEG-P(MLT) (10 mg; 25 DP; 7500 *M_w_*) was dissolved in 1 mL NMP. Chymotrypsin hydrochloride (20 mg) was dissolved in HEPES buffer (2 mL). Chymotrypsin solution (1 mL) was added to polymer solution, transferred to a dialysis bag (1000 MWCO) and dialyzed against 15 mM HEPES buffer (50 mL) containing 10 mM of CaCl_2_ and a 1% solution of penicillin and streptomycin [14]. Polymer was left shaking on a rotary shaker at 37 °C for 72 h. The dialysate was sampled at various timepoints and analyzed by HPLC to quantify free MLT released over time.

### 2.8. Cytotoxicity of PEG-P(MLT)

HEK 293 human embryonic kidney cells, RAW 264.7 murine macrophage cells and THP-1 human monocytes were seeded in 96-well plates at 2.5 × 10^4^ cells/well [16]. After 24 h, PEG-P(MLT) was added to make final concentrations of 200, 100, 50, 25, 12, 6, 3 and 1.5 μg/mL. The cell viability was determined after 48 h of incubation by adding 10 μL of CCK8 (Dojindo Laboratories, Kumamoto, Japan) into each well and culturing for 2 h at 37 °C. Cell viability was measured by the absorbance at 450 nm using a Spark multimode microplate reader (Tecan Trading AG, Männedorf, Switzerland).

### 2.9. Self-Assembly of Polymeric Micelles

The micelles were assembled from block copolymers of PEG-P(MLT) by dissolving polymers in DMSO (1 mg/mL) and self-assembled through solvent exchange by membrane dialysis (MWCO = 3500 Da) against pure water for 24 h. Micelles were then filtered through a 0.22 μm filter and characterized by dynamic light scattering (DLS). All DLS measurements reported were using a Zetasizer Nano ZS90 (Malvern Instruments) with laser wavelength of 532 nm.

### 2.10. Cell Uptake Assay

PEG-P(MLT) polymers were labeled at the terminal amino group with Alexa Fluor 647-NHS dye. Alexa Fluor 647-NHS ester solution was prepared by dissolving 1 mg of Alexa Fluor powder into 1 mL DMF. PEG-P(MLT) polymer solution was prepared by dissolving 10 mg of polymer into 1 mL of DMF. Alexa Fluor solution (100 µL) was added to the polymer solution and stirred at room temperature for 3 h under light protected conditions. Excess dye was removed by column separation using LH-20. Polymers were then self-assembled into micelles by solvent exchange by membrane dialysis and sterile filtered through a 0.22 μm filter. THP-1 cells were plated into a 12-well plate at a density of 5 × 10^5^ cells and incubated with dye labelled micelles in the presence of LPS. At 2, 4 and 24 h, cells were collected and washed by centrifugation to remove free micelles in supernatant. Cells were resuspended in 1 mL PBS with 2 μL of DAPI for 15 min to stain for dead cells. Cells were then collected and washed by centrifugation to remove free DAPI and then resuspended in PBS for analysis by flow cytometry (Becton Dickinson, Franklin Lakes, NJ, USA).

### 2.11. KYN Inhibition Assay

Digested MLT was prepared for cell culture experiments by lyophilizing dialysate (50 mL) and dissolving in water (5 mL). Calcium ions were precipitated out of solution by adding concentrated phosphate buffer (5 mL, 100 mM pH 7.4) to dialysate and storing at 4 °C for 1 h. Solution was then sterile filtered by a 0.22 µM syringe filter and 200 µL were tested by HPLC to confirm concentration of solutions. Stock solutions of MLT (10 mg/mL) was prepared by dissolving MLT into pure water and adding 0.1 M NaOH into solution dropwise until MLT fully dissolved. Stock solution of PEG-P (MLT) (10 mg/mL) were prepared by dissolving polymer into DMSO with sonication and heating. Stock solutions were added to cell culture medium with LPS (RPMI-1640, 10% FBS, 1% P/S, 1 µg/mL) for a final concentration of 23 μg/mL (100 µmol). THP-1 cells were collected by centrifugation and resuspended in stock solutions and then seeded in 12-well plates at 5 × 10^5^ cells/well [17]. Cell culture supernatant was sampled at 24 h and analyzed by an LC-EXTREMA HPLC (JASCO, Tokyo, Japan) for KYN concentration.

### 2.12. Quantification of TNF-α Expression by THP-1 by Enzyme-Linked Immunosorbent Assay (*ELISA*)

THP-1 cells were seeded at 1 × 10^6^ cells/mL in a 24-well cell culture plate and incubated with 50 ng/mL of PMA for 48 h, followed by a 24 h incubation period with RPMI-1640. THP-1 cells were then incubated with and without LPS (1 µg/mL) in the presence of MLT, PEG-P(MLT) micelles at a concentration of 23 µg/mL (100 µmol). THP-1 supernatants were removed after 24 h of culture and stored at −70 °C for the subsequent cytokine and chemokine analyses. TNF-α was indicated by color changes of the TMB solutions and measured by absorption spectrophotometer (λ = 450). The means and standard error (SE) of the means were calculated and presented in the results as the means ± SE of the means.

### 2.13. Quantification of NF-κB Expression in RAW 264.7 by Secreted Alkaline Phosphatase (SEAP) Reporter Gene Assay

RAW 264.7 macrophages transfected with a secreted alkaline phosphatase (SEAP) reporter construct inducible by NF-κB were seeded at 2 × 10^5^ cells/200 µL in 96-well cell culture plates with various concentrations of PEG-P(MLT) micelles: (0, 0.01, 0.1, 1, 5, 10, 20, 30, 40, 50 mM). After 24 h, 0.2 µg of lipopolysaccharide (LPS) were added to each well and incubated for another 24 h. SEAP concentrations were quantified using a QUANTI-Blue solution following the manufacturer’s instructions. Briefly, 20 µL of the assay solutions added to 200 µL of culture supernatant and incubated at 37 °C for 1 h. NF-κB expression was quantified by absorption spectrophotometer (λ = 620).

### 2.14. HPLC Detection

Tryptophan, MLT, MDT and KYN were detected by HPLC [18]. Chromatography was performed with a TSKgel RP C-18 (4.6 mm ID × 50 mm L, porosity 300 Å, particle size 5 μm) (Tosoh Bioscience, Tokyo, Japan) with a guard column (reversed-phase C18 column of 4.6 mm ID × 12.5 mm L, porosity 300 Å, particle size 5 μm) (Tosoh Bioscience, Tokyo, Japan) using a mobile phase containing a pH 4 acetate buffer with 27 mL/L acetonitrile. Detection for TRP, MLT and MDT was carried out by fluorescence detector at excitation: 266 nm, and emission: 360 nm. Detection for KYN was carried out by UV detector at 360 nm.

## 3. Results

### 3.1. Enzymatic Degradation of P(MLT) and P(MDT)

**Figure 2:**
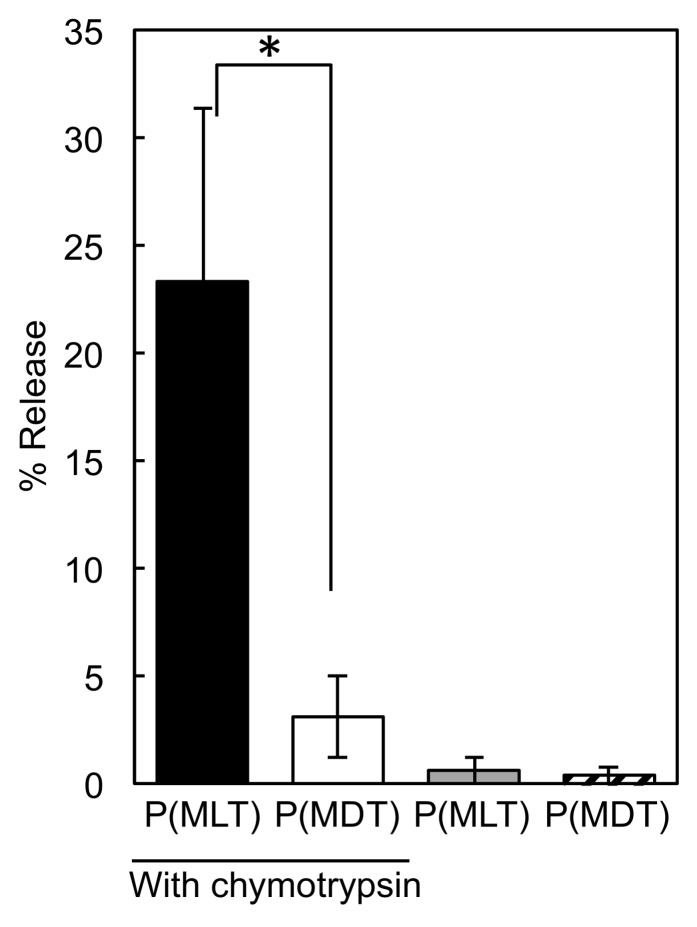
Release of small molecule IDO inhibitors from homopolymers of P(MLT) and P(MDT) with and without chymotrypsin. Data shown as the mean ± S.D. (*n* = 3). * *p* < 0.01 determined by Student’s *t*-test.

We hypothesized that amphiphilic block copolymers comprised of PEG and methyl tryptophan polymers capable of proteolytic release would be an excellent strategy for enhancing pharmacokinetics. However, the proteolytic release depends on substrate recognition of the polymers. Biological enzymes are highly specific in substrate recognition and even slight changes in chemistry are often enough to prevent enzymatic activity. In fact, D-peptides are often used in academic or pharmaceutical applications when proteolytic resistance is desired, as proteolytic enzymes do not recognize D-amino acids as substrates [14,19]. For this reason, before investing significant efforts into synthesizing amphiphilic block copolymers of controlled size and dispersity through ring opening polymerization, we synthesized P(MLT) and P(MDT) by condensation of the amino acids to determine if they could be enzymatically cleaved to release small molecule MLT and MDT. Moreover, we hypothesized that enzymes that use hydrophobic peptide residues like tryptophan or phenylalanine as substrates would be the enzymes most likely capable of digesting P(MLT) and P(MDT). Thus, chymotrypsin was selected as the enzyme for digesting the homopolymers, as chymotrypsin is a proteolytic enzyme that is selective for hydrophobic residues [20]. Additionally, as chymotrypsin is a model enzyme often used in biological studies, it has well-defined methods, protocols and kinetics for peptide bond hydrolysis. P(MLT) and P(MDT) homopolymers were synthesized through simple condensation reaction with MLT or MDT monomers and an amide coupling agent [21,22] (DMTMM). The production of polymers was confirmed by GPC and the absence of free monomer was confirmed by HPLC (Figure S1). Although the polymers were not uniform, these polymers served as a useful substrate to study the enzymatic activity of chymotrypsin. P(MLT) and P(MDT) were then incubated with and without chymotrypsin for 48 h to quantify the release of small molecule MLT or MDT, respectively (Figure 2). The results show that chymotrypsin hydrolyzed P(MLT), whereas the cleavage of P(MDT) was less efficient. In the absence of chymotrypsin, neither P(MLT) nor P(MDT) released any significant quantity of small inhibitors. These results provided a strong rationale to further develop P(MLT) as the polymeric backbone for enzymatic release of IDO inhibitors.

### 3.2. Synthesis and Characterization of NPC-MLT

**Figure 3:**
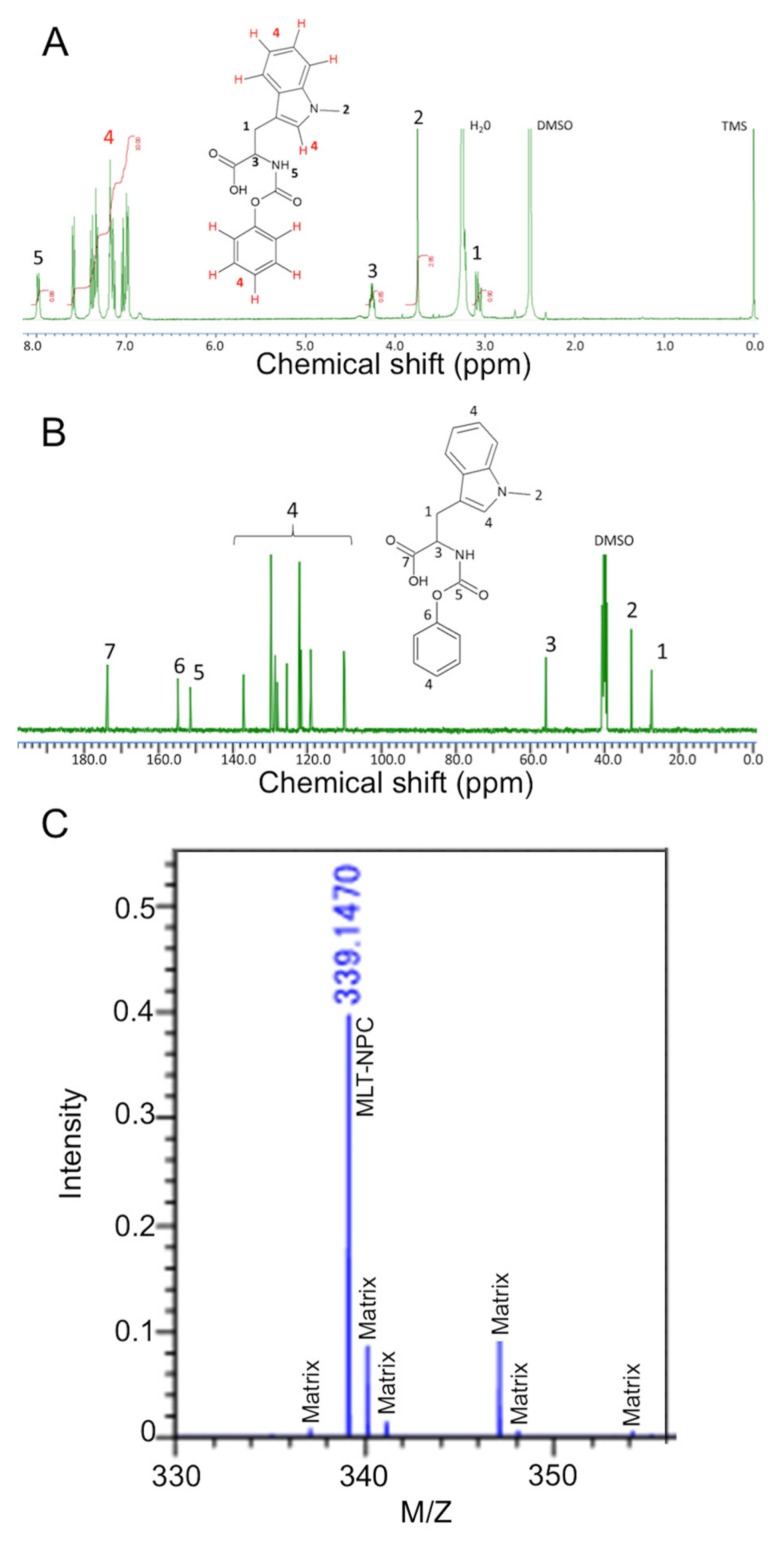
(**A**) ^1^H-NMR spectra of MLT-NPC in DMSO-d_6_. (**B**) ^13^C-NMR spectra of MLT-NPC in DMSO-d_6_. (**C**) MALDI-TOF of MLT-NPC.

The synthesis of block copolymers and poly(amino acids) with controlled size and polydispersity requires the use of ring opening polymerizations [23]. Typically, poly(amino acids) are polymerized by using *N*-carboxy anhydride (NCA) of amino acids, which are usually synthesized by using triphosgene [23]. The NPC of amino acids is a relatively new method for synthesizing poly(amino acids) through self-cyclization to generate NCA at high temperatures in situ [15,24,25,26,27]. The synthesis of NPC derivatives has significant advantages over traditional NCAs, including mild reaction conditions, improved stability and the use of less toxic reagents. In this study, MLT-NPC was successfully synthesized through carbamylation of MLT (Figure 1B) and characterized by ^1^H-NMR, ^13^C-NMR and MALDI-TOF MS (Figure 3).

The ^1^H-NMR spectra of MLT-NPC showed almost 100% carbamylation of MLT, as determined quantitatively by the integration of the aromatic peaks corresponding to MLT and the benzyl carbamate moiety (Figure 3A). 13C-NMR spectra further confirmed the structure as all the peaks could be assigned to the carbons of MLT-NPC (Figure 3B). In addition, the MALDI-TOF MS analysis of MLT-NPC revealed the presence of a single product. Thus, these results indicate the successful synthesis and purification of MLT-NPC.

### 3.3. Synthesis of PEG-P(MLT) and Self-Assembly of Micelles

**Figure 4:**
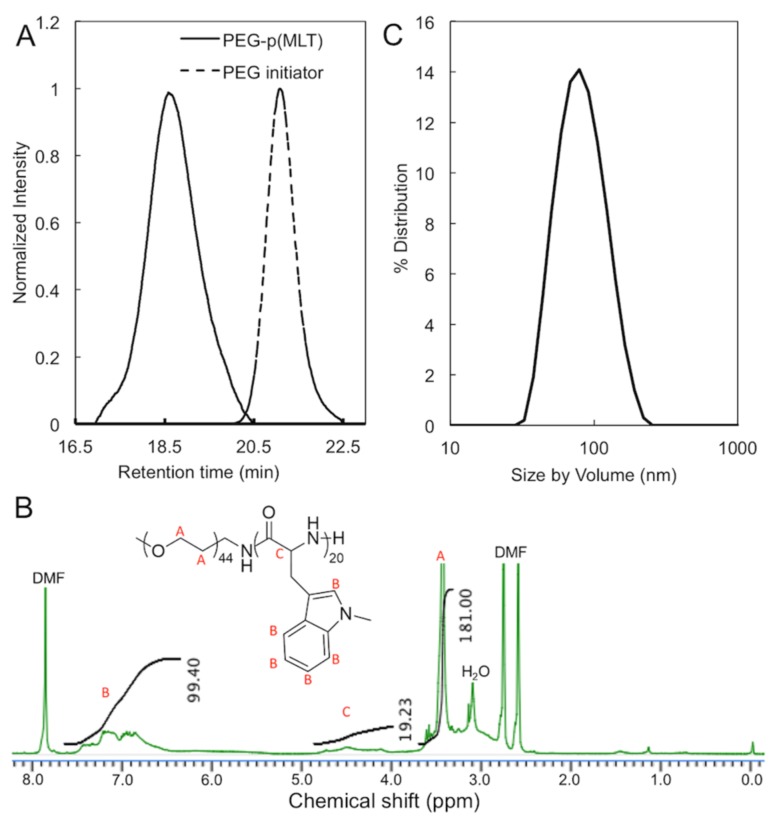
(**A**) GPC chromatogram of poly(ethylene glycol) (PEG) (*M_w_* = 2000 Da) and PEG-P(MLT). (**B**) ^1^H-NMR of PEG-P(MLT). (**C**) Dynamic light scattering (DLS) histogram obtained from self-assembly of PEG-P(MLT) in water.

The polymerization of MLT-NPC (Figure 1C) was initiated from the terminal amino group of α-methoxy-ω-amino PEG with a molecular weight of 2000. The reaction was carried out for 24 h and the polymer was collected by precipitation in ether. The final polymer was confirmed by GPC to have a molecular weight of 7000 and a polydispersity index of 1.12, indicating a degree of polymerization of 25 units of MLT per polymer. The ^1^H-NMR spectra of PEG-P(MLT) showed 20 units of MLT, as determined by the integration of the peaks corresponding to PEG (Figure 4B; peak A) and the aromatic region (Figure 4B; peaks in B).

PEG-P(MLT) was then dissolved in DMAc and dialyzed to water for self-assembling polymeric micelles. This method provided narrowly distributed polymeric micelles with a z-average diameter by volume of 80 nm and polydispersity index of 0.16, confirming the ability for PEG-P(MLT) to self-assemble into micelles (Figure 4C).

### 3.4. Release of MLT from PEG-P(MLT) after Enzymatic Cleavage

**Figure 5:**
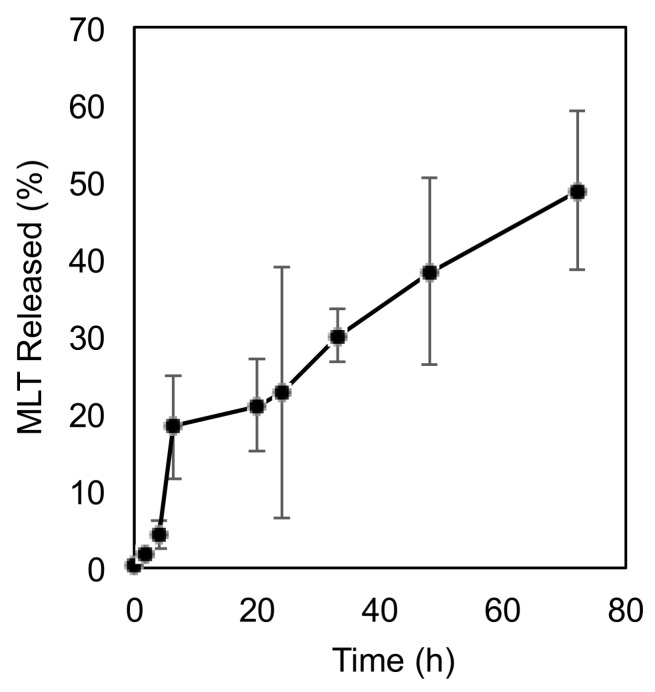
Release of small molecule MLT from PEG-P(MLT) by chymotrypsin cleavage. Data shown as the mean ± S.D. (*n* = 3).

After the successful synthesis of PEG-P(MLT) from MLT-NPC, we performed another chymotrypsin assay using the same protocols initially used for the P(MLT) and P(MDT). To confirm if PEG-P(MLT) could be enzymatically hydrolyzed to release MLT. It is important to note that PEGylation has been shown to impart proteolytic resistance to peptides and proteins, which is advantageous when developing therapeutic proteins [28]. However, in the context of enzymatic prodrugs, PEGylation may inhibit proteolytic release of PEG-P(MLT). The results show that PEG-P(MLT) can be hydrolyzed in the same conditions used for P(MLT), demonstrating that PEGylation of our P(MLT) with a 2000 PEG does not inhibit the enzymatic attack and that the polymers can release of MLT (Figure 5). Thus, PEG-P(MLT) could function as effective prodrugs for sustained release of MLT after enzymatic cleavage. 

### 3.5. Cytotoxicity of PEG-P(MLT)

**Figure 6:**
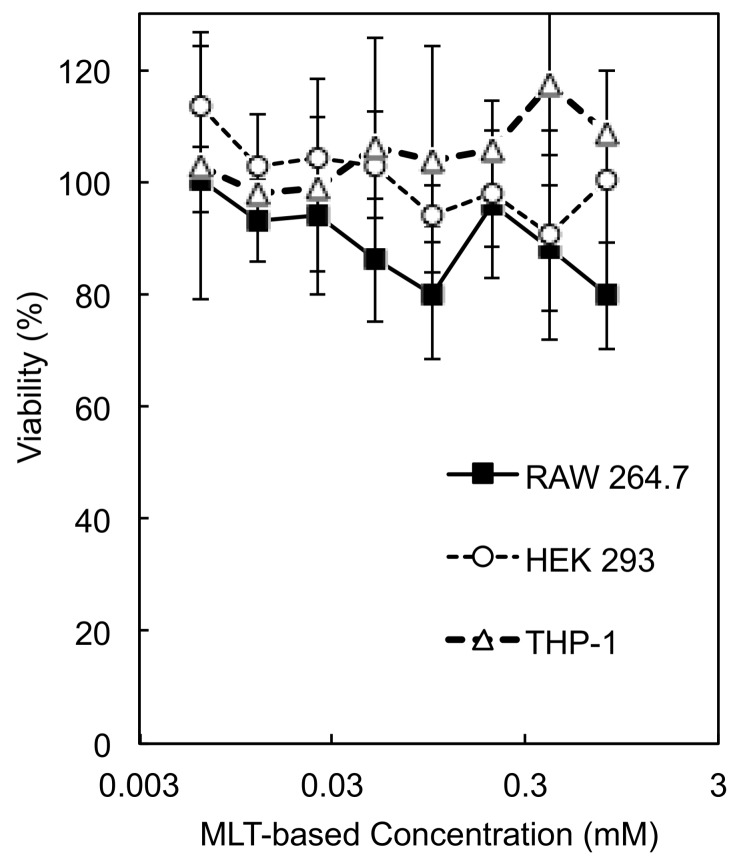
Cytotoxicity of MLT micelles against RAW 264.7 murine macrophages, HEK 293 human embryonic kidney cells and THP-1 human monocytes. Data shown as the mean ± S.D. (*n* = 8).

Next, we assessed the cytotoxicity of our block copolymer against HEK 293 cells, RAW 264.7 macrophages and THP-1 human monocytes (Figure 6). Cells were cultured in the presence of PEG-P(MLT) micelles in varying concentrations and assessed for cytotoxicity at 48 h using CCK-8. Our results show that PEG-P(MLT) are not cytotoxic against HEK 293 cells, RAW 264.7 macrophages and THP-1 human monocytes, indicating the safety of our system.

### 3.6. Cellular Uptake of PEG-P(MLT) Micelles

**Figure 7:**
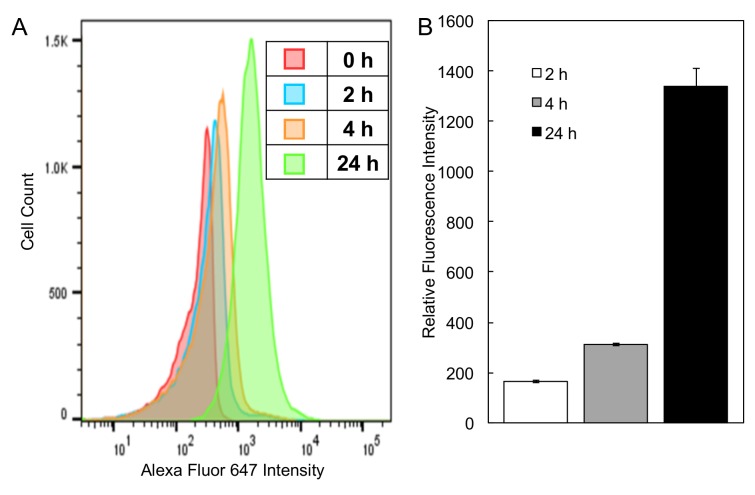
(**A**) Flow cytometry histograms of THP-1 cell uptake of Alexa Fluor 647 labelled micelles by cell population. (**B**) Graph of THP-1 cell uptake of Alexa Fluor 647 labelled micelles by mean fluorescence intensity. Data shown as the mean ± S.D. (*n* = 3).

For P(MLT) to be digested to release MLT, it must first be internalized by macrophages. To confirm cellular uptake by macrophages, PEG-P(MLT) micelles were labeled with Alexa-Fluor 647 dye and incubated with macrophages for up to 24 h. As demonstrated in Figure 7, cell uptake of dye labeled micelles increased over 24 h, confirming that micelles are taken up by THP-1 cells.

### 3.7. Inhibition of KYN by PEG-P(MLT) Micelles

**Figure 8:**
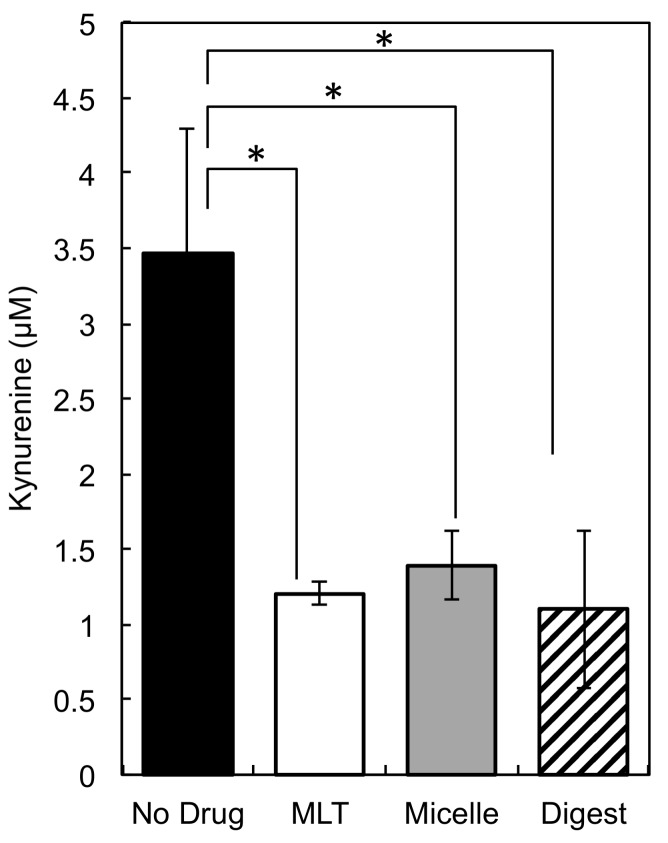
Concentration of kynurenine (KYN) in supernatant of THP-1 human monocytes after 24 h treatment with lipopolysaccharide (LPS) and MLT, micelles or chymotrypsin digested P(MLT). Data shown as the mean ± S.D. (*n* = 3). * *p* < 0.01 determined by Student’s *t*-test.

Next, we checked if the PEG-P(MLT) micelles and the polymer digests could effectively inhibit IDO in an IDO expressing cell line. For this experiment, we selected THP-1 for its high expression of IDO upon LPS stimulation [17], as well as its capacity for phagocytosis [29] and high expression of proteolytic enzymes, such as lysozyme [30]. We compared the inhibitory effect of the small molecule MLT, the PEG-P(MLT) micelles and the PEG-P(MLT) digests after chymotrypsin treatment (Figure 8). The results showed that the PEG-P(MLT) micelles have inhibitory efficacy, reducing the levels of KYN, which demonstrates that endogenous cellular proteases in THP-1 cells are capable of enzymatically releasing MLT from PEG-P(MLT). Moreover, the digest released from the PEG-P(MLT) after chymotrypsin treatment showed comparable efficacy to MLT, showing that the enzymatically degraded polymers have IDO inhibitory capacity.

### 3.8. Macrophage Activity in the Presence of PEG-P(MLT) Micelles

**Figure 9:**
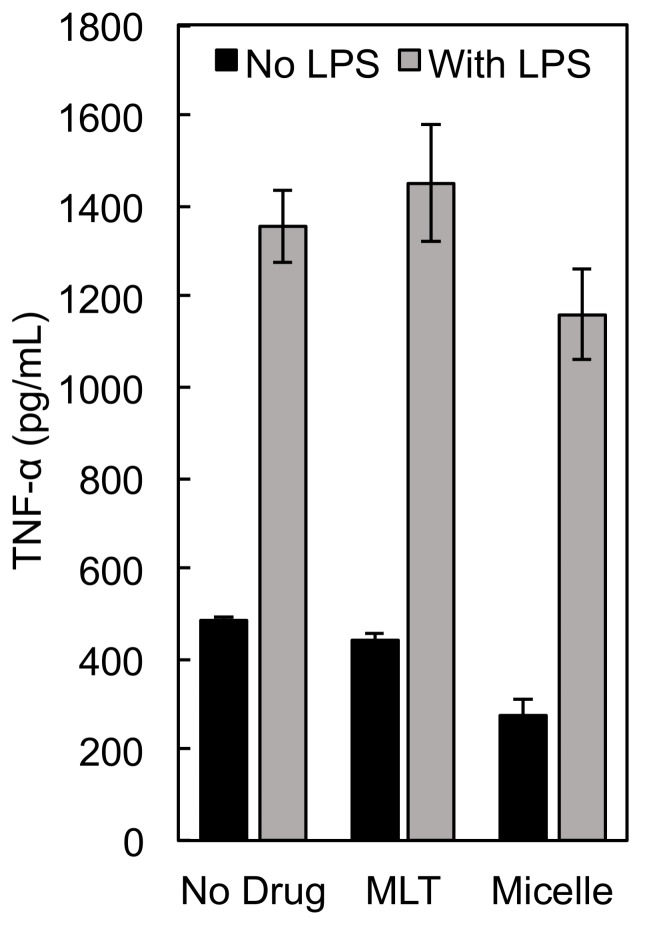
TNF-α expression levels in the presence of MLT and MLT micelles showing no increased production of TNF-α in the presence of MLT or PEG-P(MLT) micelles. Data shown as the mean ± S.D. (*n* = 3).

TNF-α is a hallmark inflammatory cytokine secreted by LPS activated macrophages that is critical for activating the adaptive immune response [31] and anti-tumor T cell immunity [32]. As shown in Figure 9, in the presence of LPS, TNF-α concentrations did not significantly change in the presence of MLT or PEG-P(MLT) micelles, suggesting that MLT has little influence on TNF-α expression. Furthermore, when not stimulated by LPS, TNF-α concentrations slightly decrease, suggesting that PEG-P(MLT) micelles alone will not activate the adaptive immune system and thus are not immunogenic.

**Figure 10:**
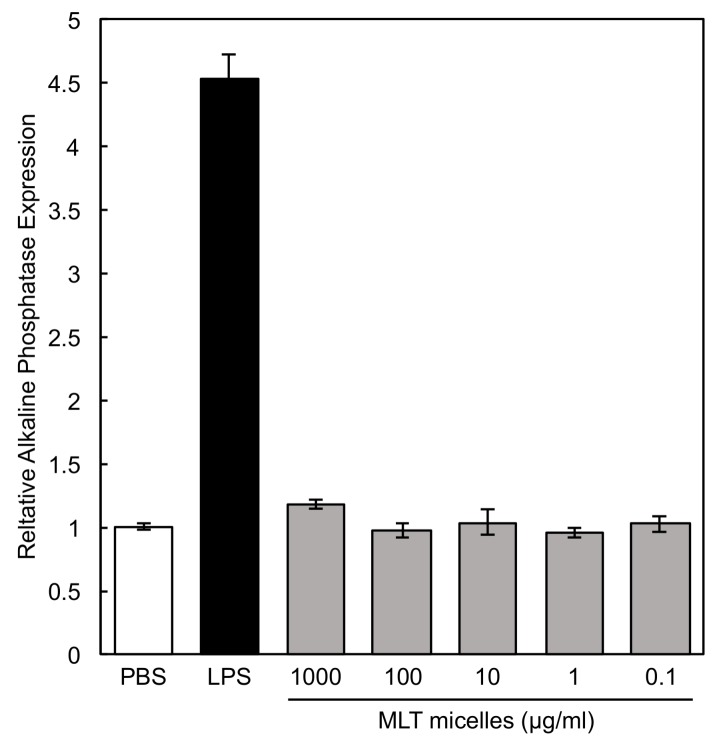
Dose dependent NF-κB expression levels measured by secreted alkaline phosphatase of RAW 264.7 macrophages in the presence of MLT micelles. LPS (1 μg/mL) was used as positive control. Data shown as the mean ± S.D. (*n* = 3).

PEG-P(MLT) micelles at different concentrations were also incubated with RAW 264.7 macrophages with a secreted alkaline phosphatase (SEAP) reporter construct inducible by NF-κB. The results confirmed that PEG-P(MLT) do not induce NF-κB at any concentration (Figure 10). As NF-κB is a DNA transcription protein that controls macrophage polarization and cytokine production, the lack of NF-κB expression suggests that PEG-P(MLT) micelles are safe and non-immunogenic.

## 4. Discussion

Our results demonstrate the ability of P(MLT)-based materials to be used as enzymatically activated IDO inhibitors. By developing a novel polymerization strategy based on NPC-MLT and PEG-NH_2_ as the macroinitiator, we were able to synthesize narrowly distributed PEG-P(MLT), which self-assembled polymeric micelles with the hydrophobic P(MLT) segment serving as the core and the PEG chains forming the shell. Thus, the micelles are designed such that the core-forming segment is made from the drug itself, reducing the nanocarrier material just to the hydrophilic PEG, a Federal Drug Administration (FDA) approved polymer often used for drug and protein conjugation. These micelles safely inhibited IDO activity, reducing the KYN production in THP-1 cells, probably due to the release of active MLT after enzymatic attack of the P(MLT) segment. These activities indicate the potential of PEG-P(MLT) micelles as IDO inhibitors directed to tumors.

The synthesis of the new monomer, NPC-MLT, was key for the polymerization of the P(MLT) segment of PEG-P(MLT). This kind of monomer and polymerization method present several advantages compared to the traditional ring opening polymerization of NCAs of amino acids [26,28]. For example, while NCA of amino acids are generally prepared by using toxic triphosgene [20] and must be kept in cool and extremely anhydrous conditions to avoid degradation, the NPC monomers can be obtained by using less toxic reagents and can be preserved in less restricted environments. Moreover, previous work has shown the possibility to synthesize poly(amino acids) with high degree of polymerization and narrow molecular weight by using NPCs [25,26,27,28,33]. The polymerization NPC-MLT from the PEG-NH_2_ resulted in a PEG-P(MLT) with a sharp molecular weight distribution, which is essential for assembling narrowly distributed polymeric micelles [12].

Polymeric micelles have shown selective accumulation in tumor tissues after systemic injection [11,13,34], which is mediated by the enhanced permeability of the tumor vasculature and the limited lymphatic drainage of tumor tissues. Thus, the accumulation levels of micelles in preclinical tumor models have been found to range from 5% up to 20% of the injected dose per gram of tumor tissue at 24–48 h after injection [34]. By considering the IC_50_ of MDT against IDO as 20 µM and a tumor accumulation of PEG-P(MLT) micelles of 5% of the injected dose per gram of tumor tissue, it can be assumed that the dose of PEG-P(MLT) micelles necessary for 50% IDO inhibition in tumors would be approximately 400 µM, or 87 mg/mL, on an MLT-basis. This dosage is considerably lower than the reported dose of MDT in the clinic, i.e., 2 g per day. Thus, it could be expected that reasonable dosages of PEG-P(MLT) micelles could promote prolonged and effective inhibition of IDO.

A hallmark of cancer is overexpression of proteases, including matrix metalloproteinases (MMPs) and cathepsins [35]. Although we demonstrated that our micelles can be recognized by the proteolytic enzyme chymotrypsin, which is an enzyme expressed in the pancreas for digestion of hydrophobic peptides [36], it is important to note that chymotrypsin-like enzymes are widely overexpressed in human cancers [37]. Moreover, proteolytic enzymes that hydrolyze hydrophobic peptides, such as cathepsin D and aminopeptidase N, have been demonstrated to be overexpressed in various tumors [38,39,40]. In addition, there are dozens of proteolytic enzymes that exist within the lysosome, a vesicular organelle responsible for hydrolyzing biomolecules [39]. Thus, it is likely that in the harsh proteolytic environment of tumors and in the lysosomes of the cells in tumors, PEG-P(MLT) would be hydrolyzed to release MLT. Such selective enzymatic activation of the PEG-P(MLT) micelles in tumor tissues could further increase the tumor specificity of the IDO inhibition.

MLT and MDT act as immunometabolic adjuvants to enhance immunotherapy by rescuing T cells from apoptosis and energy. As they do not exert antitumor effects by themselves, they are usually applied in combination with chemotherapy and immune checkpoint inhibitors [41]. Thus, our PEG-P(MLT) micelles are expected to be used together with other therapeutic drugs for synergistic efficacy. In this regard, the possibility to load hydrophobic small molecule drugs in PEG-P(MLT) micelles appears as a substantial advantage for developing synergistic therapies targeted to tumors. Our previous studies have shown that micelles prepared from amphiphilic block copolymers can stably load a wide range of hydrophobic drugs in their core and deliver them to solid tumors [10,11,42]. Particularly, the co-delivery of complementary bioactive agents within the same micelle can facilitate the simultaneous control of pharmacokinetics and the manipulation of intratumoral dosages for optimal synergy [42].

Besides the evident application of PEG-P(MLT) micelles in cancer, the micelles also have potential for developing effective approaches against immunosuppressive infections, such as tuberculosis, as well as chronic viral infections, such a HIV and hepatitis. In these diseases, IDO expression has been demonstrated to enhance immune evasion through T cell suppression [43]. In particular, combining MDT with standard of care drugs improved outcomes in primates infected with tuberculosis [44] or simian immunodeficiency virus (SIV) [45]. By loading antibiotic or antiviral drugs into PEG-P(MLT) micelles, it may be possible to enhance existing therapies through a combination of synergistic and targeted treatments for a variety of diseases.

## 5. Conclusions

We have developed a novel approach for constructing MLT-based polymeric prodrugs based on the ring opening polymerization of NPC-MLT. These polymeric prodrugs can be further exploited to generate amphiphilic block copolymers by merging with hydrophilic polymers for assembling polymeric micelles. Our results demonstrate the ability of the micelles from PEG-P(MLT) for safely inhibiting IDO activity and the subsequent KYN production, most likely by the release of MLT after enzymatic attack to the P(MLT) segment. These findings indicate PEG-P(MLT) and PEG-P(MLT) micelles as promising platforms for the development of effective tumor-targeted IDO inhibitors.

## Supplementary Materials

The following are available online at https://www.mdpi.com/2079-4991/9/5/719/s1, Figure S1: Chromatograms of homopolymer synthesized by condensation reaction.

## References

[1] Tang J., Pearce L., Donnell-tormey J.O., Hubbard-lucey V.M. (2018). FROM THE ANALYST ’ S COUCH Trends in the global immuno-oncology landscape. Nat. Publ. Gr..

[2] Prendergast G.C., Jaffee E.M. (2007). Cancer Immunologists and Cancer Biologists: Why We Didn’t Talk Then but Need to Now. Cancer Res..

[3] Rieth J., Subramanian S. (2018). Mechanisms of intrinsic tumor resistance to immunotherapy. Int. J. Mol. Sci..

[4] Munn D.H., Mellor A.L. (2016). IDO in the Tumor Microenvironment: Inflammation, Counter-Regulation, and Tolerance. Trends Immunol..

[5] Brochez L., Chevolet I., Kruse V. (2017). The rationale of indoleamine 2,3-dioxygenase inhibition for cancer therapy. Eur. J. Cancer.

[6] Soliman H.H., Minton S.E., Han H.S., Ismail-Khan R., Neuger A., Khambati F., Noyes D., Lush R., Chiappori A.A., Roberts J.D. (2016). A phase I study of indoximod in patients with advanced malignancies. Oncotarget.

[7] Ino K., Yoshida N., Kajiyama H., Shibata K., Yamamoto E., Kidokoro K., Takahashi N., Terauchi M., Nawa A., Nomura S. (2006). Indoleamine 2,3-dioxygenase is a novel prognostic indicator for endometrial cancer. Br. J. Cancer.

[8] Fox E., Oliver T., Rowe M., Thomas S., Zakharia Y., Gilman P.B., Muller A.J., Prendergast G.C. (2018). Indoximod: An Immunometabolic Adjuvant That Empowers T Cell Activity in Cancer. Front. Oncol..

[9] Hou D.Y., Muller A.J., Sharma M.D., DuHadaway J., Banerjee T., Johnson M., Mellor A.L., Prendergast G.C., Munn D.H. (2007). Inhibition of indoleamine 2,3-dioxygenase in dendritic cells by stereoisomers of 1-methyl-tryptophan correlates with antitumor responses. Cancer Res..

[10] Cabral H., Kataoka K. (2014). Progress of drug-loaded polymeric micelles into clinical studies. J. Control. Release.

[11] Cabral H., Miyata K., Osada K., Kataoka K. (2018). Block Copolymer Micelles in Nanomedicine Applications. Chem. Rev..

[12] Kataoka K., Harada A., Nagasaki Y. (2012). Block copolymer micelles for drug delivery: Design, characterization and biological significance. Adv. Drug Deliv. Rev..

[13] Maeda H., Wu J., Sawa T., Matsumura Y., Hori K. (2000). Tumor vascular permeability and the EPR effect in macromolecular therapeutics a review. J. Control. Release.

[14] Miller S.M., Simon R.J., Ng S., Zuckermann R.N., Kerr J.M. (1995). Comparison of the Proteolytic Susceptibilities of Homologous L-Amino Acid, D-Amino Acid, and N-Substituted Clycine Peptide and Peptoid Oligomers. Drug Dev. Res..

[15] Yamada S., Atsushi S., Goto M., Endo T. (2013). Facile Synthesis of Poly ( L -tryptophan ) through Polycondensation of Activated Urethane Derivatives. J. Polym. Sci. Part A Polym. Chem..

[16] Wu H., Tao A., Martin J.D., Quader S., Liu X., Takahashi K., Hespel L., Miura Y., Hayakawa Y., Irimura T. (2017). Proteasome Inhibitor e Loaded Micelles Enhance Antitumor Activity Through Macrophage Reprogramming by NF- k B Inhibition. J. Pharm. Sci..

[17] Fujigaki S., Saito K., Sekikawa K., Tone S., Takikawa O., Fujii H., Wada H., Noma A., Seishima M. (2001). Lipopolysaccharide induction of indoleamine 2,3-dioxygenase is mediated dominantly by an IFN-γ-independent mechanism. Eur. J. Immunol..

[18] Laich A., Neurauter G., Widner B., Fuchs D. (2002). More Rapid Method for Simultaneous Measurement of Tryptophan and Kynurenine by HPLC. Clin. Chem..

[19] Werle M., Bernkop-Schnürch A. (2006). Strategies to improve plasma half life time of peptide and protein drugs. Amino Acids.

[20] Hedstrom L. (2002). Serine Protease Mechanism and Specificity. Chem. Rev..

[21] HO C.H., Odermatt E., Berndt I., Tiller J.C. (2008). Ways of Selective Polycondensation of L-Lysine Towards Linear a- and e-Poly-L-Lysine. J. Polym. Sci. Part A Polym. Chem..

[22] Okamura A., Hirai T., Tanihara M., Yamaoka T. (2002). Synthesis and properties of novel biodegradable polyamides containing α-amino acids. Polymer (Guildf).

[23] Cheng J., Deming T.J., Deming T. (2012). Synthesis of Polypeptides by Ring-Opening Polymerization of α-Amino Acid N-Carboxyanhydrides BT - Peptide-Based Materials. Peptide-Based Materials.

[24] Doriti A., Brosnan S.M., Weidner S.M., Schlaad H. (2016). Synthesis of polysarcosine from air and moisture stable N-phenoxycarbonyl-N-methylglycine assisted by tertiary amine base. Polym. Chem..

[25] Yamada S., Koga K., Endo T. (2012). Useful synthetic method of polypeptides with well-defined structure by polymerization of activated urethane derivatives of α-amino acids. J. Polym. Sci. Part A Polym. Chem..

[26] Yamada S., Sudo A., Goto M., Endo T. (2014). Phosgene-free synthesis of polypeptides using activated urethane derivatives of α-amino acids: An efficient synthetic approach to hydrophilic polypeptides. RSC Adv..

[27] Yamada S., Koga K., Sudo A., Goto M., Endo T. (2013). Phosgene-free synthesis of polypeptides: Useful synthesis for hydrophobic polypeptides through polycondensation of activated urethane derivatives of α-amino acids. J. Polym. Sci. Part A Polym. Chem..

[28] Turecek P.L., Bossard M.J., Schoetens F., Ivens I.A. (2016). PEGylation of Biopharmaceuticals A Review of Chemistry and Nonclinical Safety Information of Approved Drugs. J. Pharm. Sci..

[29] Baqui A.A.M.A., Meiller T.F., Turng B.F., Kelley J.I., Falkler W.A. (1998). Functional changes in THP-1 human monocytic cells after stimulation with lipopolysacchar1de of oral microorganisms and granulocyte macrophage colony stimulating factor. Immunopharmacol. Immunotoxicol..

[30] Tsuchiya S., Yamabe M., Yamaguchi Y., Kobayashi Y., Konno T., Tada K. (1980). Establishment and characterization of a human acute monocytic leukemia cell line (THP-1). Int. J. Cancer.

[31] Trevejo J.M., Marino M.W., Philpott N., Josien R., Richards E.C., Elkon K.B., Falck-Pedersen E. (2002). TNF-α-dependent maturation of local dendritic cells is critical for activating the adaptive immune response to virus infection. Proc. Natl. Acad. Sci. USA.

[32] Calzascia T., Pellegrini M., Hall H., Sabbagh L., Ono N., Elford A.R., Mak T.W., Ohashi P.S. (2007). TNF-α is critical for antitumor but not antiviral T cell immunity in mice. J. Clin. Investig..

[33] Yamada S., Ikkyu K., Iso K., Goto M., Endo T. (2015). Facile synthesis of polymethionine oxides through polycondensation of activated urethane derivative of α-amino acid and their application to antifouling polymer against proteins and cells. Polym. Chem..

[34] Cabral H., Matsumoto Y., Mizuno K., Chen Q., Murakami M., Kimura M., Terada Y., Kano M.R., Miyazono K., Uesaka M. (2011). Accumulation of sub-100 nm polymeric micelles in poorly permeable tumours depends on size. Nat. Nanotechnol..

[35] Hanahan D., Weinberg R.A. (2011). Review Hallmarks of Cancer The Next Generation. Cell.

[36] Fruton J.S., Bergmann M. (1942). The Multiple Specificity of Chymotrypsin. J. Biol. Chem..

[37] Kondakova I.V., Spirina L.V., Koval V.D., Shashova E.E., Choinzonov E.L., Ivanova E.V., Kolomiets L.A., Chernyshova A.L., Slonimskaya E.M., Usynin E.A. (2014). BIOLOGY Chymotrypsin Like Activity and Subunit Composition of Proteasomes in Human Cancers. Mol. Biol..

[38] Garcia M., Platet N., Liaudet E., Laurent V., Derocq D., Brouillet J., Rochefort H. (1996). Concise Review Biological and Clinical Significance of Cathepsin D in Breast Cancer Metastasis. Stem Cells.

[39] Koblinski J.E., Ahram M., Sloane B.F. (2000). Unraveling the role of proteases in cancer. Clin. Chim. Acta.

[40] Wickström M., Larsson R., Nygren P., Gullbo J. (2011). Aminopeptidase N (CD13) as a target for cancer chemotherapy. Cancer Sci..

[41] Metz R., Rust S., Duhadaway J.B., Mautino M.R., Munn D.H., Vahanian N.N., Link C.J., Prendergast G.C. (2012). IDO inhibits a tryptophan sufficiency signal that stimulates mTOR A novel IDO effector pathway targeted by D -1-methyl-tryptophan. RSC Adv..

[42] Zhang J., Kinoh H., Hespel L., Liu X., Quader S., Martin J., Chida T., Cabral H., Kataoka K. (2017). E ff ective treatment of drug resistant recurrent breast tumors harboring cancer stem-like cells by staurosporine / epirubicin co-loaded polymeric micelles. J. Control. Release.

[43] Schmidt S.V., Schultze J.L. (2014). New insights into IDO biology in bacterial and viral infections. Front. Immunol..

[44] Gautam U.S., Foreman T.W., Bucsan A.N., Veatch A.V., Alvarez X., Adekambi T. (2018). In vivo inhibition of tryptophan catabolism reorganizes the tuberculoma and augments immune-mediated control of Mycobacterium tuberculosis. Proc. Natl. Acad. Sci. USA.

[45] Boasso A., Vaccari M., Fuchs D., Hardy A.W., Tsai W.-P., Tryniszewska E., Shearer G.M., Franchini G. (2009). Combined Effect of Antiretroviral Therapy and Blockade of IDO in SIV-Infected Rhesus Macaques. J. Immunol..

