# Supplementary material for: PEG-Poly(1-Methyl-l-Tryptophan)-Based Polymeric Micelles as Enzymatically Activated Inhibitors of Indoleamine 2,3-Dioxygenase
Source: Nanomaterials (Basel). 2019 May 9;9(5):719. doi: 10.3390/nano9050719 (PMC6566635; doi:10.3390/nano9050719)
Supplement: Supplementary file 1 [file nanomaterials-09-00719-s001.pdf]

Supplementary Information

PEG-Poly(1-Methyl-L-Tryptophan)-Based Polymeric Micelles as Enzymatically Activated Inhibitors of Indoleamine 2,3-Dioxygenase

George Lo Huang, Anqi Tao, Takuya Miyazaki, Thahomina Khan, Taehun Hong, Yasuhiro Nakagawa and Horacio Cabral *

Department of Bioengineering, Graduate School of Engineering, The University of Tokyo, 7-3-1 Hongo, Bunkyo-ku, Tokyo 113-8656, Japan; huang@bmw.t.u-tokyo.ac.jp (G.H.); tao@bmw.t.u-tokyo.ac.jp (A.T.); tmiyazaki@bmw.t.u-tokyo.ac.jp (T.M.); khan@bmw.t.u-tokyo.ac.jp (T.K.); hong@bmw.t.u-tokyo.ac.jp (T.H.); nakagawa@bmw.t.u-tokyo.ac.jp (Y.N.)

* Correspondence: horacio@bmw.t.u-tokyo.ac.jp

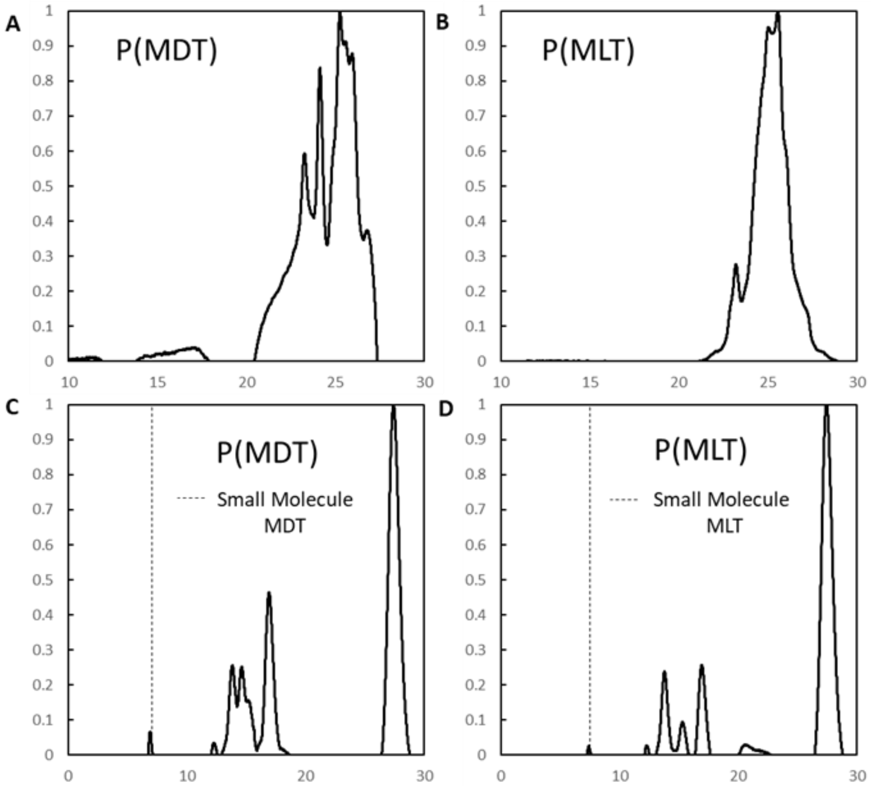

Figure S1. A, B. Size exclusion chromatography of homopolymers P(MDT) and P(MLT) prepared by condensation reaction. **C, D.** HPLC of homopolymers for the detection of trace amounts of unreacted methyl tryptophan (dotted line).
